# Bilateral Renal Hypoperfusion Following Motor Vehicle Accident

**DOI:** 10.1177/2324709618794726

**Published:** 2018-08-24

**Authors:** Medha Joshi, Salah Aldergash, Ali Hussain, Rakesh Gulati, Varun Malhotra

**Affiliations:** 1 Temple University-Conemaugh Memorial Medical Center, Johnstown, PA, USA; 2 Jefferson University Hospital, Philadelphia, PA, USA

**Keywords:** blunt trauma, bilateral renal hypoperfusion, motor vehicle accident

## Abstract

Renal hypoperfusion noted on abdominal computed tomography (CT) scan without any underlying comorbid condition is a rare finding. Most reported cases of renal hypoperfusion have an association with an underlying cardioembolic problem, such as atrial fibrillation, endocarditis, cardiomyopathies, or artificial valve thrombi. We present a case of transient renal hypoperfusion evident on abdominal CT scan following blunt trauma. An 18-year-old male without any significant past medical history presented to the emergency department with the complaint of abdominal pain. The patient reported history of motor vehicle accident 1 week prior to his presentation. He was a front seat passenger wearing a seatbelt when the car went into a ditch. Airbags were deployed and the patient briefly lost consciousness. He presented 1 week later with complaints of generalized abdominal pain, more on the left side that started a few days after his accident, nonradiating, constant, 4/10 intensity. He denied dysuria, hematuria, groin pain, fever, chills, nausea, vomiting, abdominal pain, diarrhea, constipation, decreased oral intake, joint pain, leg swelling, or redness. He denied any medication use or any history of intravenous drug abuse. There was no reported family history of kidney disease or blood clots. Initial laboratory tests, including complete blood count, basic metabolic panel, erythrocyte sedimentation rate, and urinalysis were unremarkable except trace protein on the urinalysis. Contrast-enhanced CT of the abdomen showed multiple, confluent, focal areas of hypoperfusion of the renal parenchyma bilaterally. Given the CT findings of bilateral renal hypoperfusion, the patient was admitted to the hospital and an extensive workup was performed to rule out cardioembolic etiology. Echocardiogram, renal ultrasound, magnetic resonance angiogram of the abdomen, vasculitis panel, and hypercoagulable workup was unremarkable. The CT findings of renal hypoperfusion were considered secondary to transient hypoperfusion from blunt trauma. Abdominal pain resolved with nonsteroidal anti-inflammatory drugs and he was discharged to home. Follow-up abdominal CT scan with contrast obtained a few months later showed normal kidneys with resolution of previously noted renal hypoperfusion. Our case highlights a benign incidental finding of bilateral renal hypoperfusion following motor vehicle accident (with airbag injury), which resolved on follow-up imaging. On literature search, such CT scan findings of transient renal hypoperfusion of unclear significance have not been previously reported. Even though our patient underwent extensive workup to rule out cardioembolic etiology, it may be reasonable to forego such workup following blunt abdominal trauma.

## Introduction

Bilateral renal hypoperfusion detected on abdominal computed tomography (CT) scan without any underlying comorbid condition is a rare finding. Most reported cases of renal hypoperfusion have an association with an underlying cardioembolic problem, such as atrial fibrillation, endocarditis, cardiomyopathies, or artificial valve thrombi. We present a case of transient renal hypoperfusion evident on abdominal CT scan following blunt trauma. To our knowledge, this is the first such reported case.

## Case

An 18-year-old male without any significant past medical history presented to the emergency department with the complaint of abdominal pain. Pain described as generalized abdominal pain, more on the left flank that started 5 days ago, nonradiating, constant, 4/10 intensity. He denied dysuria, hematuria, groin pain, fever, chills, nausea, vomiting, abdominal pain, diarrhea, constipation, decreased oral intake, joint pain, leg swelling, or redness. He denied any medication use or any history of illicit drug use. The patient reported history of motor vehicle accident (MVA) 1 week prior to his presentation. He was a front seat passenger wearing a seatbelt when the car accidently went into a ditch. Airbags were deployed and patient briefly lost consciousness.

**Figure 1.**
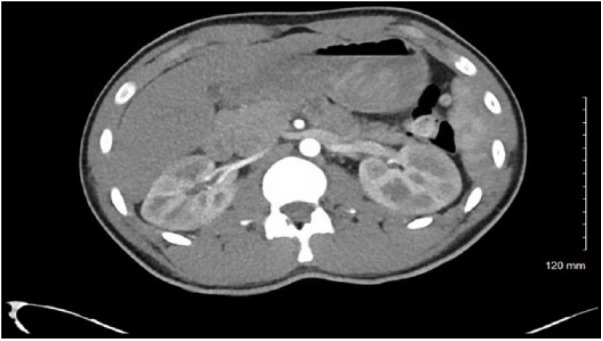
Bilateral renal hypoperfusion following motor vehicle accident.

There was no reported family history of kidney disease or blood clots. Physical examination revealed left flank tenderness but no evidence of ecchymosis. Laboratory tests including complete blood count, basic metabolic panel (BUN [blood urea nitrogen] 20 mg/dL, creatinine 1.1 mg/dL), sedimentation rate, urine drug screen, and complete urinalysis were unremarkable, except trace proteinuria without evidence of microscopic hematuria. Contrast-enhanced CT (CECT) of the abdomen was performed as no diagnosis was clear on clinical evaluation. CECT showed multiple, confluent, focal areas of hypoperfusion of the renal medulla and cortices bilaterally (Figure 1). Given the CT findings of bilateral renal hypoperfusion, the patient was admitted to the hospital and an extensive workup was performed to rule out cardioembolic etiology. Transthoracic echocardiogram and renal ultrasound were unremarkable. Hypercoagulable workup including prothrombin time, partial thromboplastin time, dilute Russell viper venom test screen, fibrinogen level, antithrombin III activity, protein C activity, protein S antigen, and prothrombin gene mutation was unremarkable. Mild D-dimer elevation was noted, 285 ng/mL (normal 0-250 ng/mL). Vasculitis panel including ANA, ANCA, complement levels, rheumatoid factor, and hepatitis serology was unremarkable.

**Figure 2.**
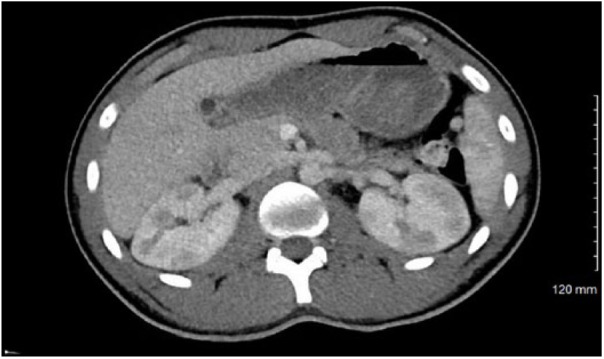
Magnetic resonance angiography of the abdomen—normal renal circulation.

Magnetic resonance angiography of the abdomen revealed widely patent renal arteries and normal perfusion in all branches of abdominal aorta without evidence of thrombosis (Figure 2). The CT findings of renal hypoperfusion were considered secondary to transient hypoperfusion from blunt trauma. Abdominal pain was considered musculoskeletal in origin due to blunt trauma, which resolved with ibuprofen and he was discharged to home.

**Figure 3.**
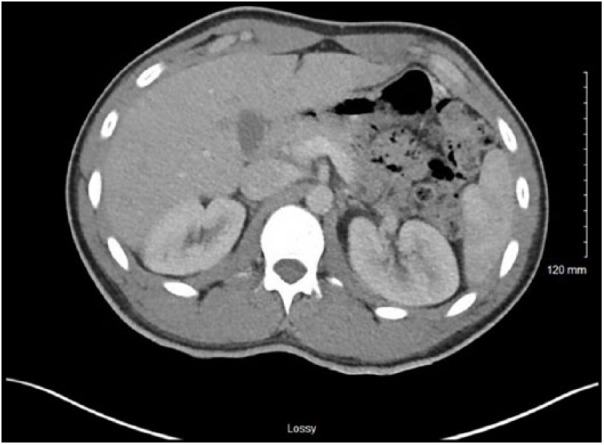
Normalized renal perfusion on follow-up imaging.

The patient was followed up as an outpatient a few weeks after discharge. He was asymptomatic with a normal physical examination. On follow-up testing, renal function was normal (BUN 9 mg/dL, creatinine 0.9 mg/dL). Complete urinalysis was unremarkable, with resolution of previously noted trace proteinuria. Follow-up abdominal CT scan with contrast showed normal kidneys with complete resolution of previously noted renal hypoperfusion (Figure 3).

## Discussion

Approximately 10% of all significant blunt abdominal traumatic injuries manifest with involvement of the kidneys, although it is usually minor.^1^ MVAs are the most common cause resulting in sudden deceleration or crush injuries that may affect the renal parenchyma or the vascular pedicle.^1-3^ The kidney is particularly vulnerable to deceleration injuries (eg, falls, motor vehicle collisions) because it is fixed in space only by the renal pelvis and the vascular pedicle. Flank ecchymosis and broken ribs are signs suggestive of renal injury. CT scan with intravenous (IV) contrast enhancement including delayed imaging remains the most common method of evaluating for extravasation of urine from the collecting system.^4^ CT with IV contrast is the imaging modality of choice. It has supplanted IV pyelogram. IV pyelogram may be useful in select cases, such as suspected ureteral injury where delayed CT images are nondiagnostic. Ultrasound has limited usefulness in cases of testicular trauma and torsion and bladder injury.

Our case highlights a benign incidental finding of bilateral renal hypoperfusion following MVA (with airbag injury), which resolved on follow-up imaging. On literature search, such imaging findings of transient renal hypoperfusion have not been previously reported.

The American Association for the Surgery of Trauma grading system^5^ is the most commonly used injury grading system for renal trauma. The classification describes various elements of renal trauma, grades I to V, ranging from contusion to laceration (Table 1). Grade IV injuries include segmental infarctions without associated lacerations,^1^ deceleration resulting in stretching and tearing of the intima, with the resultant intimal flap causing thrombosis has been described as the proposed mechanism.^6^ Complete infarction is less common than segmental or subsegmental infarction in patients sustaining blunt trauma.^3^

**Table 1.** AAST Kidney Injury Scale.^5^

Grade^a^	Type of Injury	Description of Injury
I	Contusion	Microscopic or gross hematuria, urologic studies normal
	Hematoma	Subcapsular, nonexpanding without parenchymal laceration
II	Hematoma	Nonexpanding perirenal hematoma confirmed to renal retroperitoneum
	Laceration	<1.0 cm parenchymal depth of renal cortex without urinary extravagation
III	Laceration	<1.0 cm parenchymal depth of renal cortex without collecting system rupture or urinary extravagation
	Laceration	Parenchymal laceration extending through renal cortex, medulla, and collecting system
IV	Vascular	Main renal artery or vein injury with contained hemorrhage
V	Laceration	Completely shattered kidney
	Vascular	Avulsion of renal hilum, which devascularizes kidney

Abbreviation: AAST, American Association for the Surgery of Trauma.

a Advance one grade for bilateral injuries up to grade III.

A case series identified 106 patients with recreationally acquired renal injuries, among which majority (85%) of the injuries were snow sport related. The renal trauma appeared to follow different patterns, mostly without associated organ injuries or hypotension; however, transient hypoperfusion was not reported.^7^

The American Urological Association issued a guideline on urotrauma in 2014 and updated it in 2017.^4^ Recommendations regarding renal trauma included the following: in stable blunt trauma patients with gross hematuria or microscopic hematuria and systolic blood pressure <90 mm Hg, diagnostic imaging with IV CECT should be performed. Diagnostic imaging with IV CECT should be performed in stable trauma patients whose mechanism of injury or physical examination findings raise concern for renal injury (eg, rapid deceleration, significant blow to the flank, rib fracture, significant flank ecchymosis, penetrating injury of the abdomen, flank, or lower chest). IV contrast-enhanced abdominal/pelvic CT with immediate and delayed images should be performed when renal injury is suspected.

Our patient’s CT findings did not reveal evidence of renal infarction or thrombosis. He had bilateral renal hypoperfusion that was incidentally detected 1 week following abdominal blunt trauma associated with MVA. These findings were not associated with hematuria or abnormal kidney function. Renal perfusion defects completely resolved on follow-up imaging after a few months. The exact mechanism of these findings appears unclear. We suspect abdominal blunt trauma with sudden deceleration during MVA may have contributed to bilateral medullary-cortical hypoperfusion that was incidentally noticed on imaging 1 week following MVA.

Even though our patient underwent extensive workup to rule out cardioembolic etiology, it may be reasonable to forego such workup following blunt abdominal trauma in an otherwise healthy, young individual with no associated comorbidities.
